# Assessment of oral health and cost of care for a group of refugees in Germany: a cross-sectional study

**DOI:** 10.1186/s12903-018-0535-1

**Published:** 2018-04-27

**Authors:** Katja Goetz, Wiebke Winkelmann, Jost Steinhäuser

**Affiliations:** Institute of Family Medicine, University Hospital Schleswig-Holstein, Campus Luebeck, Ratzeburger Allee 160, 23538 Luebeck, Germany

**Keywords:** Cost of care, Dental screening, Oral health, Refugees

## Abstract

**Background:**

There is a research gap concerning the evaluation of the oral healthcare of refugees. Therefore, the aim of this study was to evaluate the oral health of refugees and to estimate the costs of oral care.

**Methods:**

The study was conceptualized as a pilot study. The study participants were refugees who lived either in collective living quarters or at a reception center in a region of the federal state of Schleswig-Holstein, Germany. The cross-sectional design was complemented by dental screening. Data were collected from August 2016 until July 2017. The basic condition of the teeth was evaluated using a convenience sample by a single dentist. The assessment of caries was carried out visually in accordance with the International Caries Detection and Assessment System from code 3 and higher. The DMF-T (decayed, (D), missing, (M), filled (F), teeth (T)) index was calculated. The costs of oral care were analyzed for conservative treatment (filling or extraction) and for prosthetic treatment (missing teeth) in the form of a bridge or crown.

**Results:**

The dental screening was attended by 102 refugees, with a mean age of 28 years. A total of 49% of the study sample suffered from toothache, and the DMF-T index had a mean of 6.89. For 92% of the study sample, treatment was indicated, and a cost estimate of the treatment could be calculated. The average cost of conservative treatment was estimated to be 205.86 EUR, and the average cost of prosthetic treatment was estimated to be 588.0 EUR. The oral healthcare costs of the different treatment procedures were higher for refugees that presented with toothache than for those without toothache, with the exception of prosthetic treatment procedures.

**Conclusions:**

There is a lack of population-based data that survey the oral health status of refugees. Therefore, the current study presents an initial overview regarding the oral health status and the potential costs of oral healthcare of refugees.

**Electronic supplementary material:**

The online version of this article (10.1186/s12903-018-0535-1) contains supplementary material, which is available to authorized users.

## Background

Oral health is essential for well-being and is a quality-of-life determinant [1]. There is a strong relationship between oral health and general health. For example, severe periodontal disease is associated with type 2 diabetes [2]. Moreover, periodontal disease and dental caries are the two most prevalent oral afflictions worldwide [2]. Effective prevention of these diseases is related to access to oral healthcare, periodic assessment of oral health and individual oral health behavior [2]. Therefore, it may be assumed that refugees are a high-risk group for developing oral disease because of their limited access to dental care. Various factors, such as limited access to oral healthcare, oral care products and to nutritious food and clean water could lead to poor oral health status, including the development of periodontal disease or dental caries. Moreover, factors such as access to and organization of healthcare in the country of origin could contribute to the poor oral health of refugees, which is shown in the oral health database provided by the World Health Organization (WHO) [3].

By the end of 2016, there were approximately 22.5 million refugees and 2.8 million asylum seekers worldwide [4, 5]. Over 55% of these refugees originate from South Sudan, Afghanistan, and Syria countries with human rights violations [4, 5]. In Europe, there were approximately 5.2 million refugees, and 669,500 refugees had entered Germany by the end of 2016 [5]. In Germany, the asylum policy has no limit regarding the entry of refugees. The entitlements to medical care by refugees in Germany are detailed in the Asylum-Seekers’ Benefits Act (AsylbLG), and §4, 6 AsylBLG specifically regulates the access to health care, which is limited [6, 7]. These laws detail what types of health problems allow for access to healthcare. According to §4 AsylBLG, these constitute acute diseases and pain that requires medical or dental treatment [6].

Different systematic reviews show a research gap concerning the evaluation of the oral healthcare of refugees [8, 9]. Therefore, the aim of this study was to evaluate the oral health of refugees in one region in Germany and to estimate the costs of oral care on the basis of regular and restricted access.

## Methods

The currents study was conceptualized as a pilot study with a cross-sectional design.

### Design and participants

This study complies with the Strengthening the Reporting of Observational Studies in Epidemiology (STROBE) guidelines [10]. The study participants were refugees who lived in either collective living quarters or at a reception center in one region of the federal state of Schleswig-Holstein, Germany. The participants were recruited to the project “Mobile Medical Practice” (Rollende Arztpraxis). The “Rollende Arztpraxis” was financed by the Damp-Stiftung and provided general practice care for refugees in rural regions of Schleswig-Holstein [11]. In connection with this project, dental screening was offered in the “Rollende Arztpraxis” in a non-dental setting. This was a small bus that was furnished with equipment such as a treatment table, a chair, an emergency electrocardiogram, a spirometer, a defibrillator and a pocket Doppler. The dentist used the treatment table for dental screenings, enabling the refugees to lie down so that a more effective evaluation of the oral health status could be obtained. The dentist used magnifying glasses with light, a plane mouth mirror and a dental probe for the screening.

The examination of oral health was performed by a dentist supported by interpreters who provided translation in different languages. The following languages could be translated by the interpreters: Arabic, Farsi, Persian and Russian. Three interpreters were available, which was in agreement with the facility manager of the collective living quarters and reception center. The dental screening service was announced in advance so that the interpreters could be arranged.

A calibration of the dentist was not addressed because it is not part of the recommendations included in the STROBE guidelines [10].

A dentist in the region was recommended if a participant suffered from pain or the screening showed the need for dental treatment. The data were collected from August 2016 until July 2017.

After verbal and written information was given by the interpreter, written informed consent was obtained from each participant. Participation in the study was completely voluntary.

### Measurements

The measurement process consisted of a two-step procedure. First, sociodemographic data were collected, and then the dental screening was carried out.

#### Sociodemographic data

The sociodemographic data included gender, age, country of origin, number of months residing in Germany and the location where they were currently living. This questionnaire was in the German language and was translated by the interpreter. The questionnaire was a separate step, decoupled from the oral health screening.

#### Evaluation of oral health

Information regarding oral health was obtained from different questions, such as the date of the last visit to a dentist, daily dental hygiene, access to dental hygiene products and the presence or absence of toothache. All questions were in German and were translated by the interpreter for the refugee. The toothache pain level was determined by a visual analogue scale ranging from 0 (no pain) to 10 (extremely strong pain). The condition of the teeth was evaluated by a dentist. Dental caries were assessed visually in accordance with the International Caries Detection and Assessment System from code 3 and higher [12]. The tools used for the oral health screening were as follows: magnifying glasses with light, a plane mouth mirror and a dental probe. The results were entered into a paper-based schematic that depicted all 32 teeth, including five areas in the posterior region (mesial, occlusal, distal, buccal and oral), five areas in the front tooth region (mesial, distal, incisal, labial and oral), and the root and tooth necks. The questionnaire of the evaluation of the oral health was added as Additional file 1.

### Data analysis

Data analysis was performed using SPSS 24.0 (SPSS Inc., IBM). Continuous data were summarized using the mean and standard deviation. Categorical data were presented as frequency counts and percentages. Student’s t-test with case exclusion was used for the comparison between toothache (yes/no) and healthcare costs (conservative, prosthetic and overall costs). Additionally, refugees who were in need of a removable denture were considered as a fixed denture within the analysis because this is covered by standard care.

#### Calculation of the DMF-T index

The DMF-T (decayed, (D), missing, (M), filled (F), teeth (T)) index was calculated. With this index, it is possible to assess the patient’s individual risk of caries. All teeth, excepting the wisdom teeth, were included in the calculation to obtain the sum score, which ranged from 0 to 28. A score of 0 indicates that all teeth are without decay, and no teeth are missing, filled or destroyed. Furthermore, the DMF-T index can be divided into four categories: less than 5.0 indicates a very low risk of caries, 5.0 to 8.9 indicates a low risk of caries, 9.0 to 13.9 indicates a moderate risk of caries, and greater than 13.9 indicates a high risk of caries [13].

#### Methodology of healthcare cost estimation

The estimation of costs was based on destroyed and decayed teeth that required conservative treatment in the form of tooth filling or extraction and on missing teeth that required a prosthetic treatment in the form of a bridge or crown. The costs of oral healthcare were estimated using two different German statutory health insurance plans and were averaged. The calculated costs included payments that would be reimbursed in a regular care setting, material costs and treatment costs. An overview of the estimated costs for the different treatment procedures is provided in Table 1.

**Table 1:** Methodology to estimate oral healthcare costs (in EUR)

	Procedure	Mean of costs (in EUR)
Conservative treatment: Destroyed teeth		
Lower jaw (one root)	Block anesthesia	11.62
	Extraction	9.68
	Overall cost	21.30
Lower jaw (≥ two roots)	Block anesthesia	11.62
	Extraction	14.52
	Overall cost	26.14
Upper jaw (one root)	Infiltration anesthesia	7.75
	Extraction	9.68
	Overall cost	17.43
Upper jaw (≥ two roots)	Infiltration anesthesia	7.75
	Extraction	14.52
	Overall cost	22.27
Conservative treatment: Decayed teeth		
Parts of lower jaw	Block anesthesia	11.62
	Indirect pulp capping	5.81
	Areas of filling (min)	31.00
	Areas of filling (max)	56.14
	Overall cost (min/max)	48.43 / 73.57
Upper jaw	Infiltration anesthesia	7.75
	Indirect pulp capping	5.81
	Areas of filling (min)	31.00
	Areas of filling (max)	56.14
	Overall cost (min/max)	44.56 / 69.70
Prosthetic treatment: Crown	Full cast	143.27
	Unveneered	152.94
	Overall cost	296.21
Prosthetic treatment: Bridge	Full cast	114.23
	Vestibular veneer	124.00
	Pontic	60.00
	Overall cost	298.23

### Ethical approval

Ethical approval for this research study was obtained from the University of Luebeck in June 2016 (Approval No. 16–123). Participation in the study was voluntary. No additional data were evaluated.

## Results

One hundred and two refugees and asylum seekers participated in the pilot study and attended a dental screening. The total number of refugees residing in the participating collective living quarters and reception center is unknown. Therefore, no response rate can be calculated. Table 2 presents the sociodemographic characteristics of the participants. More than 80% of the responding refugees were male. The mean age was 28.6 years (SD = 10.3). Participants were from nine different countries; over 24% migrated from Afghanistan, 18% migrated from Iraq, and 14% migrated from Syria.

**Table 2:** Sociodemographic data of the study sample (*n* = 102)

Characteristic		Number
Months residing in Germany, mean (SD);range		13.9 (5.6) 3–24
Age, mean (SD);range		28.6 (10.3) 16–64
Gender, n (%)	Female	18 (17.6%)
	Male	84 (82.4%)
Residence, n (%)	Reception center	21 (20.6%)
	Collective living quarters	81 (79.4%)
Country of origin, n (%)	Afghanistan	25 (24.5)
	Iraq	19 (18.6)
	Syria	15 (14.7)
	Eritrea	14 (13.7)
	Yemen	11 (10.8)
	Armenia	7 (6.9)
	Somalia	5 (4.9)
	Iran	4 (3.9)
	Chechnya	2 (2.0)

SD standard deviation

### Oral health of refugees

The evaluation of oral health is presented in Table 3. Over 84% of the study participants did not visit a dentist regularly during their childhood. Nearly 50% of the participants practiced dental hygiene twice per day. The presence of toothache afflicted 49% of the participants, with a mean of 4.51 (SD = 1.9) on the pain scale. The DMF-T index had a mean of 6.89 (SD = 5.5). Healthy dentition was present in 13.7% of the participants. A moderately high DMF-T index (> 9.0) was observed in 25.5% of the refugees.

**Table 3:** Oral health and DMF-T index of refugees

Variable		Number (%)
Year of last dental visit	2017	31(30.4%)
	2016	29 (28.5%)
	2015	4 (3.9%)
	2014	9 (8.8%)
	< 2014	26 (25.5%)
Regular visits to a dentist during childhood	No	86 (84.3%)
	Once per year	11 (10.8%)
	Twice per year	5 (4.9%)
Daily dental hygiene	Once per day	45 (44.1%)
	Twice per day	50 (49.0%)
	More than twice per day	7 (6.9%)
Was there a time without access to dental hygiene products?	No	47 (46.1%)
	Yes	55 (53.9%)
Do you currently have a toothache?	No	52 (51.0%)
	Yes	50 (49.0%)
Toothache on the pain scale^a^, mean (SD); range		4.51 (1.9); 2–10
DMF-T index, mean (SD)		6.89 (5.5)
Categorization of DMF-T Index, n (%)	No index (0.0)	14 (13.7%)
	Very low Index (< 5.0)	38 (37.3%)
	Low Index (5.0–8.9)	24 (23.5%)
	Moderate Index (9.0–13.9)	14 (13.7%)
	High Index (> 13.9)	12 (11.8%)

^a^ranged from 0 (no pain) to 10 (extremely strong pain), SD standard deviation

The following means and standard deviations were observed for the individual DMF-T components: for decayed teeth (DT), a mean of 2.90 (SD = 2.04); for missing teeth (MT), a mean of 3.88 (SD = 2.95); and for filled teeth (FT), a mean of 3.76 (SD = 2.94).

### Estimation of oral healthcare costs

For 92% (*n* = 94) of the study population, some form of treatment was indicated. The estimated healthcare costs of the study population were divided into conservative treatment and prosthetic treatment and are presented in Table 4. Conservative treatment in cases requiring the extraction of destroyed teeth was indicated for 77 participants, and the mean cost was calculated to be 82.64 EUR (SD = 62.65). The healthcare costs for conservative treatment were calculated on the basis of 93 participants with a minimum mean of 157.47 EUR (SD = 106.0) and a maximum mean of 205.86 EUR (SD = 153.20). Prosthetic treatment was indicated for 44 participants, and the mean cost was calculated to be 588.0 EUR (SD = 395.77).

**Table 4:** Conservative and prosthetic treatment – estimation of oral healthcare costs (in EUR)

Conservative treatment		Mean (SD), range (in EUR)
Destroyed teeth (*n* = 77)		82.64 (62.65), 17.43–359.21
Decayed teeth (*n* = 61)	Minimum	135.77 (93.57), 44.56–412.65
	Maximum	209.54 (144.84), 69.70–638.91
Overall costs for conservative treatment (*n* = 93)	Minimum	157.47 (106.0), 17.43–508.50
	Maximum	205.86 (153.20), 17.43–734.76
Prosthetic treatment		Mean (SD), range
Crown (*n* = 4)		183.90 (81.3), 143.27–305.88
Bridge (*n* = 43)		587.90 (390.88), 228.46–1680.92
Overall costs for prosthetic treatment (*n* = 44)		588.0 (395.8), 143.27–1680.92
Overall oral healthcare costs (*n* = 94)	Minimum	431.03 (445.94), 22.27–1747.65
	Maximum	487.91 (463.54), 22.27–1973.91

SD standard deviation, n number of refugees who require treatment

Table 5 shows the comparison of toothache (yes/no) and healthcare costs. The healthcare costs for the different treatment procedures were greater for refugees with toothache than for those without toothache, with the exception of prosthetic treatment procedures. A significant difference was found for conservative treatment procedures, with an average difference of 46.9 EUR (minimum) and 74.6 EUR (maximum). For the overall oral healthcare, an average difference of 102.5 EUR (minimum) and 129.0 EUR (maximum) was found.

**Table 5:** Comparison of oral healthcare costs for refugees with and without toothache (in EUR, using Student’s t-test with case exclusion)

Treatment procedures		Toothache	Mean (SD) (in EUR)	Average difference	*p*-value
Cost for conservative treatment	Minimum	Yes (*n* = 48)	180.2 (106.3)	46.9	0.03
	Minimum	No (*n* = 45)	133.3 (101.2)		
	Maximum	Yes (*n* = 48)	242.0 (155.6)	74.6	0.02
	Maximum	No (*n* = 45)	167.3 (142.3)		
Cost for prosthetic treatment		Yes (*n* = 26)	572.2 (380.5)	−38.6	0.75
		No (*n* = 16)	610.8 (427.1)		
Overall oral healthcare costs	Minimum	Yes (*n* = 49)	480.1 (440.8)	102.5	0.27
	Minimum	No (*n* = 45)	377.6 (450.2)		
	Maximum	Yes (*n* = 49)	540.7 (453.6)	129.0	0.18
	Maximum	No (*n* = 45)	411.7 (469.9)		

SD standard deviation

## Discussion

Our results present an initial overview of oral health status and the potential costs of oral healthcare of refugees in one region in the federal state of Schleswig-Holstein, Germany. The study was conceptualized as a pilot study to obtain preliminary data on the oral health status and costs of oral healthcare of refugees. The sociodemographic data obtained in the study concerning the origin of the refugees were comparable to the official statistics of Schleswig-Holstein, which show that Afghanistan, Iraq and Syria are the primary countries from which people immigrate [14]. Moreover, our results concerning the distribution of gender show a high proportion of male refugees, which coincides with another study on the oral healthcare of refugees [15]. There are multiple reasons for the gender imbalance. The official statistics from the Federal Agency of Migration and Refugees show that more men than women are refugees [16]. It can also be assumed that social values and standards, such as traditional gender roles and culture in the country of origin could influence the utilization of healthcare [17]. However, more information is needed to explain why men are more likely than women to utilize oral healthcare services, and this question should be investigated in further studies.

The evaluation of oral health revealed a low level of preventive behavior during childhood. Over 84% of the participants did not visit a dentist during their childhood. The professional recommendation is to visit a dentist at least once per year [18]. When accounting for the global health workforce statistics, the low number of dentists in countries such as Afghanistan, Iraq and Syria is likely to be an important barrier to oral healthcare access and therefore to adequate prevention [19]. However, it is well known that preventive dental visits in childhood influence one’s oral health in adulthood [20]. Our study sample presented a DMF-T index of 6.9, which is lower than that of the general population in Germany (11.2 for young adults) [21]. However, a comparison of the individual DMF-T components between the refugees and the general population showed similar results, with a lower proportion of DT and a higher proportion of MT and FT for the refugee population [21]. Similar results were also obtained in a different study on the oral healthcare of refugees in Germany [15]. Furthermore, our findings are comparable to a study from Australia that revealed that the oral health status of refugees is poorer than that of the general population [22]. Oral health status depends not only on access to care but also on the social status of an individual [21]. One possible explanation for the data could be that our study participants have a relatively high to moderate social status. Additional research would be helpful to clarify how the social status of refugees affects their oral health.

A dental care report by one statutory health insurance organization indicated that 8.9% of the insured adult population has had an extraction [23]. This is in contrast to our sample, where an extraction was recommended for 75.5% of the refugees. However, if the refugees were not experiencing pain, then no treatment was offered, according to the definition of restricted access in §4 AsylBLG [6]. This restricted access for refugees could have an impact on healthcare costs. The reimbursed costs for health insurance will be lower due to the restricted access, as not all refugees need treatment, and therefore, they will not incur any cost. However, the direct and indirect costs cannot be estimated. In our sample, 50 refugees stated that they currently suffered from toothache. In these cases, §4 AsylBLG would grant them treatment, and it would be more expensive than if there was no access limitation regarding toothache. Postponing treatment until there is pain is not preventive action. Therefore, it can be assumed that restricted access may lead to discrimination of refugee’s healthcare, and it may also lead to higher costs [24]. Moreover, it is possible that persons who have poor oral health, such as missing teeth, might have problems obtaining employment, which is an important part of the integration process for refugees in a foreign society [25].

### Limitations

The number of participants was rather small. Participation in the dental screening was voluntary; therefore, there may have been a potential selection bias. Moreover, the number of how many refugees were asked for participation was unknown. It is possible that refugees who did not have any oral health problems were less likely to seek oral healthcare, and this should be considered when interpreting our results. Furthermore, it is not possible to link the sociodemographic data and the oral health status of the refugees. Therefore, a regression analysis with adjustments to evaluate the potential effects of age, gender and country of origin on oral health status was not possible and should be confirmed in further studies. The questionnaire that was used to evaluate oral health was not based on the questionnaire used in the German oral healthcare study [21]. Moreover, potential bias may have been present in the dental screening process because only one dentist screened the refugees, and no additional dentist was available for verification of the screening results. It was not possible to follow up with the refugees regarding whether or not they contacted the recommended dentists in the region and what type of treatment they received. Additionally, no assessment of the periodontium or oral mucosa was performed because of a lack of resources, such as limited time, finances and personal equipment with which to carry out the dental screening process. However, such an assessment should be performed in further studies.

Calculating a cost estimate for the different treatment procedures is quite difficult because all of the existing statutory health insurances (*n* = 113) in Germany vary in their level of reimbursed costs [26]. The healthcare costs presented in this study are hypothetical because of the restricted healthcare access of the refugees. The true costs cannot be calculated. Finally, this was a cross-sectional study; therefore, we cannot draw causal links from these findings.

## Conclusions

There is a lack of population-based data that survey the oral health status of refugees. It should be considered whether an oral health screening could be part of an initial medical examination. Standardized tools for oral health assessments are available from the WHO that could be used by advanced-stage dental students. These assessments would aid in the prevention of oral disease.

## Additional file

Additional file 1: Questionnaire “Evaluation of oral health status” The questionnaire for the presented survey is available as additional file. (PDF 94 kb) (PDF 94 kb)

## Abbreviations

AsylbLG: Asylum-seekers’ benefits act
DMF-T index: Decayed, missing, filled-teeth index
DT: Decayed teeth
EUR: Euro
FT: Filled teeth
MT: Missing teeth
SD: Standard deviation
STROBE: Strengthening the reporting of observational studies in epidemiology
WHO: World Health Organization
