# Supplementary material for: Assessment of oral health and cost of care for a group of refugees in Germany: a cross-sectional study
Source: BMC Oral Health. 2018 Apr 27;18:69. doi: 10.1186/s12903-018-0535-1 (PMC5921436; doi:10.1186/s12903-018-0535-1)
Supplement: Supplementary file 1 — Questionnaire “Evaluation of oral health status” The questionnaire for the presented survey is available as additional file. (PDF 94 kb) (PDF 94 kb) [file 12903_2018_535_MOESM1_ESM.pdf]

Evaluation of oral health status

1. When was the last visit of a dentist? ___________________

2. Did you visit a dentist during childhood regular?

- ☐ No
- ☐ Yes, once per year
- ☐ Yes, twice per year

3. How often do you clean (dental hygiene) your teeth per day?

- ☐ Never
- ☐ once per day
- ☐ twice per day
- ☐ more than twice per day

4. Was there a time without access to dental hygiene products?

- ☐ Yes
- ☐ No

5. Have you at the moment toothache?

- ☐ Yes
- ☐ No

How strong are your toothache? (0 “no pain” to 10 “extremely strong pain”)

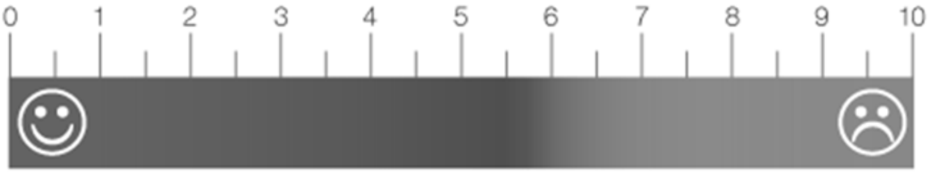

Institute of Family Medicine, University Hospital Schleswig-Holstein, Campus Luebeck, Germany I PD Dr. Katja Goetz

Dental screening

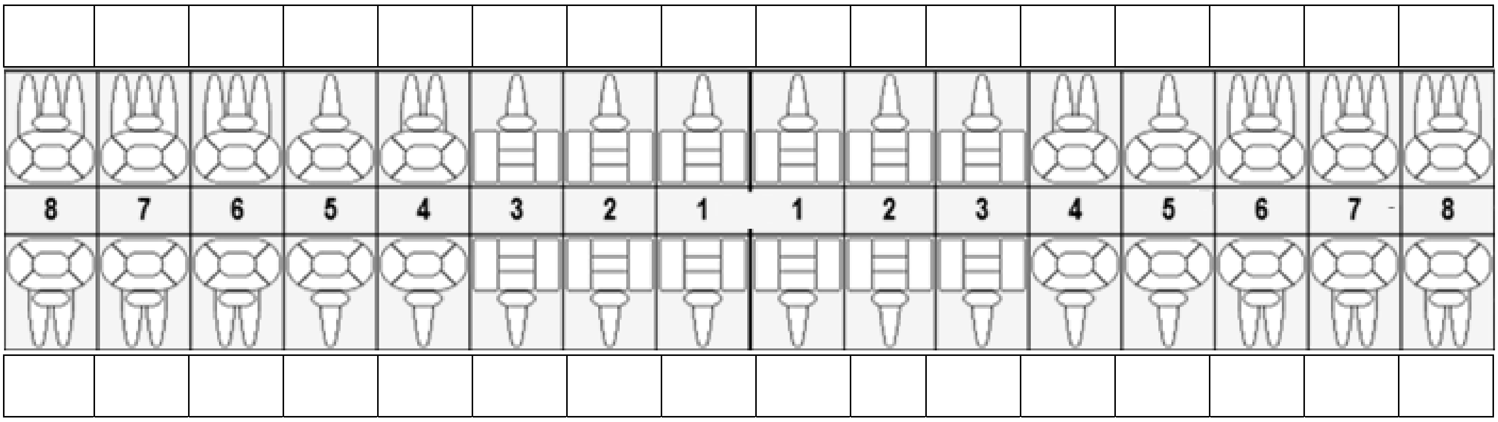

F	Filled	C	Decayed tooth (caries)
K	Crown	F	Missing tooth
B	Bridge element	X	Destroyed tooth
TK	Crown (partial)	WF	Root canal filling
I	Implantat		
E	Tooth replacement		
